# Synergistic activity and mechanism of action of *Stephania suberosa* Forman extract and ampicillin combination against ampicillin-resistant *Staphylococcus aureus*

**DOI:** 10.1186/s12929-014-0090-2

**Published:** 2014-09-11

**Authors:** Yothin Teethaisong, Nongluk Autarkool, Kittipot Sirichaiwetchakoon, Pongrit Krubphachaya, Sajeera Kupittayanant, Griangsak Eumkeb

**Affiliations:** School of Pharmacology, Institute of Science, Suranaree University of Technology, Nakhon Ratchasima, 3000 Thailand; School of Biology, Institute of Science, Suranaree University of Technology, Nakhon Ratchasima, 3000 Thailand; School of Physiology, Institute of Science, Suranaree University of Technology, Nakhon Ratchasima, 3000 Thailand

**Keywords:** β-lactam antibiotics, Ampicillin-resistant *S. aureus* (ARSA) *Stephania suberosa* Forman, Synergistic activity, Ampicillin

## Abstract

**Background:**

Ampicillin-resistant *S. aureus* (ARSA) now poses a serious problem for hospitalized patients, and their care providers. Plant-derived antibacterial that can reverse the resistance to well-tried agents which have lost their original effectiveness are the research objectives of far reaching importance. To this aim, the present study investigated antibacterial and synergistic activities of *Stephania suberosa* extracts (SSE) against ARSA when used singly and in combination with ampicillin.

**Results:**

The majority chemical compounds of SSE were alkaloid (526.27 ± 47.27 mg/1 g of dried extract). The Minimum inhibitory concentration (MICs) for ampicillin and SSE against all ARSA strains were >512 μg/ml and 4 mg/ml, respectively. Checkerboard assay revealed synergistic activity in the combination of ampicillin (0.15 μg/ml) and SSE (2 mg/ml) at fractional inhibitory concentration index (FICI) <0.5. The killing curve assay had confirmed that the viability of ARSA was dramatically reduced from 5x10^5^ cfu/ml to 10^3^ cfu/ml within 6 h after exposure to SSE (2 mg/ml) plus ampicillin (0.15 μg/ml) combination. Electron microscopic study clearly revealed that these ARSA cells treated with this combination caused marked morphological damage, peptidoglycan and cytoplasmic membrane damage, and average cell areas significant smaller than control. Obviously, Immunofluorescence staining and confocal microscopic images confirmed that the peptidoglycan of these cells were undoubtedly disrupted by this combination. Furthermore, the CM permeability of ARSA was also increased by this combination. Enzyme assay demonstrated that SSE had an inhibitory activity against β-lactamase in concentrations manner.

**Conclusions:**

So, these findings provide evidence that SSE has the high potential to reverse bacterial resistance to originate traditional drug susceptibility of it and may relate to three modes of actions of SSE: (1) inhibits peptidoglycan synthesis, resulting in morphological damage, (2) inhibits β-lactamases activity, and (3) increases CM permeability. It is widely recognized that many types of drugs are derived from alkaloids. So, this SSE offers the prominent potential to develop a novel adjunct phytopharmaceutical to ampicillin for the treatment of ARSA. Further active ingredients study, toxicity of it, and the synergistic effect on blood and tissue should be performed and confirmed in an animal test or in humans.

## Background

Since Alexander Fleming discovered the first antibiotic, an increased emergence of multidrug resistance in pathogenic bacteria has been globally documented. In London, 38% of *Staphylococcus aureus* strains were resistant to penicillin and increased to approximately 90% in the UK and USA in the recent year [1]. Similarly, 70-80% of *S. aureus* strains in most of Asian countries were resistant to methicillin [2]. *S. aureus* infections are notorious etiological pathogens for several human ailments, including pneumonia, meningitis, toxic shock syndrome, bacteremia, wound sepsis, osteomyelitis, and endocarditis [3–5]. This strain is also the second most common cause of bloodstream infection with case fatality rates of approximately 15-25% [6]. Methicillin-resistant *S. aureus* (MRSA) is notably one of the greatest threats to human health, and is the major causes of both hospital and community-acquired MRSA infection that affects both hospitalized patients and their health care providers [7,8]. Methicillin, like ampicillin, is the one member of penicillins. So, the resistance pattern of *S. aureus* against both drugs are almost the same [9]. β-lactam antibiotics are the most common and effective agents for treating staphylococcal infections, but current higher resistance levels were reported in these strains. A raised incidence of β-lactam resistance in MRSA has rapidly emerged due to mediated by acquisition of *mecA* encoded an additional penicillin-binding protein 2a (PBP2a) resulting in reducing the ability of β-lactam in binding to its target site. In summation, the production of *blaZ* encoded β-lactamase that can hydrolyze and inactivate β-lactam antibiotic activity, in particular penicillins. This enzyme is also one of the most important resistant mechanisms to β-lactam in this strain [10–12]. For these problems, searching and development of novel antibacterial compounds or new strategies are urgently required. Plants are well known and being interested sources for new antibacterial agents because they produce an enormous variety compounds to protect themselves from plant pathogens and environmental pathogens [13]. Thus, plant-derived antibacterials are often a source of novel therapeutics, but they have weaker antibacterial activity compared to that of antibiotic generated by bacteria or fungi. Therefore, it should be considered to use in a different paradigm, including combination with conventional antibiotic that has been resisted by bacteria to achieve synergism in treatment of bacterial infections [14].

Phytochemical and antibiotic combination approach has been recommended and tested by several reports for combating multidrug-resistant bacteria by achieving multiply synergistic drug targets, interacting with drug-resistant mechanisms of bacteria, and neutralizing and eliminating adverse effects [15–18]. Previous research found that the hasubanalactam alkaloid (glabradine) isolated from *Stephania glabra* showed antimicrobial activity against *Staphylococcus aureus*, *S. mutans*, *Microsporum gypseum*, *M. canis* and *Trichophyton rubrum* greater than those of novobiocin and erythromycin with IZD values of 19–27 cm [19]. To develop novel plant-derived antibacterials, the *Stephania suberosa* was selected to investigate its antibacterial property. *S. suberosa* belongs to Genus *Stephania* and family Menisperamaceae. Plants in this genus have been traditionally used for treatments of asthma, tuberculosis, dysentery, hyperglycemia, cancer, fever, intestinal discomforts, sleep disturbances and inflammation. Also, previous research found that the chemical compounds in this plant were alkaloids such as, (+)-Cepharanthine and its derivative, stephasubimine and etc. [20]. Recently, however, limited study is available on antibacterial activity of *S. suberosa*. Hence, the purpose of this investigation was to test the antibacterial activity and synergism with ampicillin of this plant against ARSA. Some elementary mechanism of actions, such as cytoplasmic membrane (CM) permeabilization, enzyme assay, and transmission electron microscopy, was also studied.

## Methods

### Plant preparation and extraction

Roots of *S. suberosa* were purchased from Lamtakhong Research Station, NakhonRatchasima Province, Thailand. The plant specimens were deposited at the Thailand National Herbarium and authenticated in comparison with the voucher specimen no. BK257189 by Dr. Paul J. Grote, a lecturer and plant biologist at Institute of Science, Suranaree University of Technology, Thailand. The roots of *S. suberosa* were cleaned thoroughly and dried under a hot air oven at the 50°C until dry. The dried samples were powdered using a grinder. Then, dried powder was extracted with 95% ethanol using Soxhlet extractor at 75°C for 8 h. The extracts were concentrated using a rotary evaporator and were lyophilized to yield a brown powder and a dark brown sticky oil of ethanolic extract.

Preliminary qualitative phytochemical screening analysis was proceeded for notable bioactive compounds, such as alkaloids, tannin, flavonoids, saponins, glycosides, steroids, terpeniods, coumarins and anthocyanin. The methods were achieved in accordance with previous description and slightly modifications [21–24]. Screening for the presence of the total alkaloid content in the extract was also accordingly carried out with previously reported [25]. In brief, 1 g of the dried extract was alkalinized with NH_4_OH, partitioned with chloroform, and evaporated to dryness on a water bath at 60°C. The net mass of triplicate remaining solid was accurately weighed and expressed as milligrams per 1 g of dried extract. After weighing, the solids were redissolved by 95% ethanol and confirmed in the presence of alkaloids.

### Bacterial strains, antibiotics, chemicals, and antibodies

Clinical isolates of ampicillin-resistant *S. aureus* (ARSA) DMST 20651, 20652 and 20653 were obtained from the Department of Medical Science, National Institute of Health, Ministry of Public Health, Thailand. The reference strain of *S. aureus* ATCC 29213 was obtained from the American Type Culture Collection (ATCC). Ampicillin, *o*-nitrophenol-β-D-galactoside (ONPG), nisin, NH_4_OH, chloroform and β-lactamase type IV from *Enterobacter cloacae* were obtained from Sigma-Aldrich, UK. Mueller-Hinton broth was purchased from Oxoid (Basingstoke, UK). Primary mouse monoclonal anti-*S. aureus* peptidoglycan antibody (ab20002), secondary goat polyclonal anti-mouse IgG conjugated with Alexa Fluor®488 (ab150117), and fluoroshield mounting medium with DAPI (ab104139) were purchased from Abcam, UK.

### Bacterial suspension standard curve

In order to obtain a known viable count of bacterial suspension, the method of Liu and company was followed as previously described with some modifications [26]. Mueller-Hinton agar and Cation-adjusted Mueller-Hinton broth (CAMHB) were practiced as a culture medium [27].

### Minimum inhibitory concentration (MIC) determinations

MIC determinations of ampicillin, nisin, and SSE against ARSA strains were performed following the method of Liu et al. [26]; Eumkeb et al. [28] and Clinical and Laboratory Standards Institute [27]. Briefly, bacterial suspension was adjusted to approximately 1 × 10^8^ cfu/ml and serial tenfold dilution was performed to achieve 5 × 10^6^ cfu/ml. The diluted inoculum (0.1 ml) of each stain was added to 0.9 ml of CAMHB plus serial dilutions of the antibacterial agents, to give a final concentration approximately 5 × 10^5^ cfu/ml. Antibiotics used and SSE was prepared by dissolving in sterile distilled water to obtain stock solutions at 1024 μg/ml for antibiotics or 1024 mg/ml of the extract. Then, the stock was serially twofold diluted to achieve respective concentration. The lowest concentration that showed no visible growth after incubated at 35°C for 18 h was recorded as the MIC. *S. aureus* ATCC 29213 was used as a reference strain.

### Checkerboard determination

The interaction between SSE and ampicillin against ARSA was assayed using checkerboard determination following Eumkeb et al. [28] and Bonapace et al. [29]. Briefly, the cultured and antibacterial agents were prepared and performed similarly with MIC determination. Otherwise, the SSE and ampicillin were combined and incubated at 35°C for 18 h. The MICs were determined as the lowest concentration SSE in combination with ampicillin. The FIC index (FICI) was calculated to determine drug interaction, and interpreted as follows:

$$ \mathrm{FIC}\ \mathrm{in}\mathrm{dex}={\mathrm{FIC}}_A+{\mathrm{FIC}}_B=\frac{\mathrm{Conc}.\ \mathrm{of}\ \mathrm{A}\ \mathrm{in}\ \mathrm{MICs}\ \mathrm{of}\ \mathrm{A}+\mathrm{B}}{\mathrm{MIC}\ \mathrm{of}\ \mathrm{A}\ \mathrm{alone}}+\frac{\mathrm{Conc}.\ \mathrm{of}\ \mathrm{B}\ \mathrm{in}\ \mathrm{MICs}\ \mathrm{of}\ \mathrm{A}+\mathrm{B}}{\mathrm{MIC}\ \mathrm{of}\ \mathrm{B}\ \mathrm{alone}} $$

Where, FICI ≤ 0.5 denoting synergistic; FICI > 0.5–4.0 denoting no interaction; FICI > 4.0 denoting antagonism [30].

### Killing curve determination

Killing curve determination was carried out in order to confirm antibacterial and synergistic activities of SSE when used singly and in combination with ampicillin. The viabilities of drug resistant bacteria after exposure to these agents alone and in combination at nine distinct times (0, 0.5, 1, 2, 3, 4, 5, 6 and 24 h) were counted. The assay was followed the previously described with some modifications [28,31]. Concisely, inocula (5 × 10^5^ cfu/ml) were exposed to SSE either singly or in combination with ampicillin. Aliquots (0.1 ml) of each exposed time were removed and diluted in normal saline as needed to enumerate 30–300 colonies. The diluted cultures were platted and spread thoroughly on plates containing MHA. After incubating at 35°C for 18 h, the growing colonies were counted. The lowest detectable limit for counting is 10^3^ cfu/ml. The experiment was performed in triplicate; data are shown as mean ± SEM.

The preliminary mechanism of action was performed in duplicate methods for confirmation except for enzyme assay that clearly determine by one method.

### Transmission electron microscopy (TEM)

Ultrastructure damages of ARSA treated with SSE either alone or in combination with ampicillin were examined using TEM. TEM preparations were performed in accordance with previously reported with slight modifications [32]. After preincubated at 35°C for 18 h, ARSA strains were adjusted spectrophotometrically to give a final concentration approximately 5 × 10^5^ cfu/ml. The cultured were grown in the absence of antibacterial agent (control), in SSE alone, ampicillin alone, and SSE plus ampicillin combination, for 4 h with shaking 110 oscillations/min in a water bath at 37°C. Then, the cultured were harvested by centrifugation at 6000 ×g for 15 min at 4°C and the pellets were fixed in 2.5% glutaraldehyde (Electron Microscope Sciences; EMS) in 0.1 M phosphate buffer (pH 7.2) for 12 h. The samples were then carefully washed twice with 0.1 M phosphate buffer. Post-fixation was carried out with 1% osmium tetroxide (EMS) in 0.1 M phosphate buffer (pH 7.2) for 2 h at room temperature. After washing in the buffer, the samples were gently dehydrated with graded ethanol (20%, 40%, 60%, 80% and 100%, respectively) for 15 min. Then, infiltration and embedding were performed using Spurr’s resin (EMS). The samples were sectioned using an ultramicrotome with a diamond knife and were then mounted on copper grids. Ultimately, the ultrathin sectioned were counterstained with 2% (w/v) uranyl acetate for 3 min and then 0.25% (w/v) lead citrate for 2 min. After staining, the specimens were viewed in a Tecnai G2 electron microscope (FEI, USA), operating at 120 kV.

In addition, the cell area of these cells from micrographs were calculated by measuring cell width multiplied by cell length (nm^2^) in order to confirm the effects of SEE either used singly and in combination on cell size.

### Immunofluorescence staining and confocal microscopy

Disruption of peptidoglycan after exposure to SSE either used singly or in adjunction with ampicillin was carried out by the immunofluorescence and visualized under a confocal laser scanning microscope. The 18 h cultured of ARSA was challenged with distinct agents; ampicillin (256 μg/ml), SSE (2 mg/ml), ampicillin (0.11 μg/ml) plus SEE (1.5 mg/ml) for 4 h. The cell grown without antibacterial agent was used as a control. After incubation, the cells were harvested by centrifugation and subsequently fixed with 2.6% paraformaldehyde and 0.04% glutaraldehyde mixture for 10 min at room temperature, and 50 min on ice. Fixed cells were washed and resuspended in PBS, smeared directly to poly-L-lysine coated slides, and air-dried. Nonspecific antibody binding in the samples was blocked with 5% BSA for 30 min at room temperature. The specimens were consecutively incubated with the primary antibody (1:800 dilution with PBS containing 2% BSA), a mouse anti-*S. aureus* peptidoglycan antibody, in a moist chamber for 1 h. The cells were washed thoroughly with PBS containing 0.1% Tween 20. The secondary antibody (Alexa 488-conjugated goat anti-mouse IgG) was prepared by diluting with PBS plus 2% BSA solution (1:1000) and incubated with the samples for 1 h in the dark at room temperature, several washed with PBS + 0.1% Tween 20. To reduce photobleaching and to counterstain bacterial DNA, the slides were mounted with a few drops of fluoroshield mounting medium containing 4′,6-Diamidino-2-Phenylindole (DAPI) [33]. Images were captured and performed with a confocal laser scanning microscope (Nikon 90i A1R) equipped with 100x NA 1.40 oil objective (Nikon), Intensilight fiber illuminator (Nikon) and NIS Elements 4.11 AR/BR B871 (Nikon). DAPI and Alexa 488 were excited at 360 nm and 488 nm, respectively. The background cell fluorescence was subtracted. An Adobe Photoshop CS5 was used for the figure preparation.

### Cytoplasmic membrane (CM) permeability

SSE used either singly or in combination with ampicillin induced CM permeability were examined by the ability of these antimicrobial agents to disclose cytoplasmic β-galactosidase activity in bacteria using ONPG as a substrate. ONPG can be cleaved by β-galactosidase localized within the cytoplasm. The products of β-galactosidase-ONPG reaction were galactose (colorless) and *o*-nitrophenol (yellow). The assays were prepared in according to the methods of Marri et al. and Eumkeb et al. with slight modification [34,35]. Shortly, 18 h ARSA cultured was adjusted to 5x10^5^ cfu/ml and grown in CAMHB without antibacterial agents (control), 2 mg/ml SSE, 256 μg/ml ampicillin and 1.5 mg/ml SSE plus 0.11 μg/ml ampicillin in 110 oscillations/min in shaking water bath at 37°C. These bacterial cells were then compiled to analyze cytoplasmic membrane alteration at six different interval times (0, 1, 2, 3, 4 and 5 h). Nisin (8 μg/ml) was applied as a positive command. Each sample 2 ml aliquots at each time were transferred to tubes containing ONPG (4 mg/ml) plus Phosphate buffered saline (PBS). Observed yellow was recorded as positive β-galactosidase activity (increased CM permeability), while appearing colorless was recorded as negative β-galactosidase activity (no effect on CM permeability).

Apart from this, the second cytoplasmic membrane permeabilization experiment was executed to confirm as previously described by Shen et al. [36] and Zhou et al. [37] with some modifications. This method was performed by measurement the release of UV-absorbing material concentrations using UV–VIS spectrophotometer. In brief, the ARSA cultures were prepared on CAMHB for 18 h at 35°C. Inocula of 2.0 ml of culture were added into 98.0 ml CAMHB and shaking at 100 r.p.m. at 37°C for 4 h to give log phase. Bacterial cultures were adjusted in saline to give 5 x 10^6^ cfu/ml. Log phase of the adjusted cultures 1.0 ml was added to 9.0 ml of 2.5 mmol/l sodium HEPES buffer (pH 7.0) supplemented with 100 mmol/l glucose plus 256 μg/ml ampicillin, 2 mg/ml SSE (½ MICs), and 0.11 μg/ml ampicillin plus 1.5 mg/ml SSE (¾ FIC) in each flask to give a final concentration at 5 × 10^5^ cfu/ml. The flasks of cell suspensions without antibacterial agent were used as the negative control and with 8 (μg/ml) nisin (½ MIC) was applied as positive control. The bacterial suspensions were incubated at 37°C in the shaker water bath. CM permeability was determined after a contact time of 0, 0.5, 1.0, 2.0, 3.0 and 4.0 h. After treatment, samples (1.0 ml) were taken every contact time and filtered through a sterile nitrate cellulose membrane (0.22 μm), and OD_260_ value of the supernatant was taken as a percentage of the extracellular UV-absorbing materials released by cells. All the measurements were done in triplicates in Varian Cary 1E UV/VIS spectrophotometer [28].

### Enzyme assay

The ability of SSE when used alone to deteriorate β-lactamase type IV activity of *E. cloacae* was performed following the method as previously described by Richards et al. [32] with little modifications. Concisely, benzylpenicillin, a substrate for β-lactamase type IV, was adjusted to concentrations sufficient to hydrolyze 50-60% substrate within 5 min. SSE at 1, 2, and 4 mg/ml were preincubated with enzyme in 50 mM sodium phosphate buffer (pH 7.0) at 37°C for 5 min prior to adding a substrate. Time-course assay were performed in 0, 5, 10, 15 and 20 min using methanol/acetic acid (100:1) as a stopping agent. 10 μl of each sample was injected to reverse-phase HPLC to analyze the remaining benzylpenicillin. A mobile phase employed was 10 mM ammonium acetate (pH 4.5 acetic acid): acetronitrile (75:25) with flow rate 1 ml/min, UV detector at 200 nm, Ascentis C18 column, and 35°C for column temperature [9]. The quantity of remaining benzylpenicillin was calculated by comparing the area under the chromatographic curve.

### Statistical analysis of experimental data

All experiments were carried out in triplicate; data were expressed as mean ± standard error of the mean (SEM) due to it takes into account sample size. Significant differences of cell area in each treated group from TEM, CM permeability and enzyme assay among each treated group at the same interval times were analyzed by one-way ANOVA. A *p* valve <0.01 of Tukey’s HSD post-hoc test was considered as statistically significant difference.

## Results

### Phytochemical screening, MIC, and checkerboard determinations

The preliminary phytochemical characteristics results of SSE indicated that the presence of alkaloids, tannins, glycosides, steroids, and anthocyanin were detected. The total alkaloid containing in the extract was 526.27 ± 47.27 mg/1 g of dried extract. The MIC results of SSE, ampicillin, and nisin against ARSA were 4 mg/ml, >512 μg/ml, and 16 μg/ml respectively, while these agents against susceptible *S. aureus* strain was 4 mg/ml, 0.25 μg/ml, and 0.5 μg/ml respectively (Table 1). According to CLSI, these outcomes suggested that ARSA used in this study revealed high resistant to ampicillin and nisin, but susceptible to reference strain *S. aureus* ATCC 29213. SSE exhibited little inhibitory effect against these strains. In checkerboard assay, based upon FICI calculation, the combination of SSE and ampicillin exhibited synergistic activity at FICI <0.5 (Table 1). Obviously, the concentration of ampicillin that can inhibit ARSA growth had considerably reduced from >512 μg/ml to 0.15 μg/ml in combination with SSE.

**Table 1:** **MICs and FICI of SSE, AMP when used either alone or in combination against ARSA**

**Strains**		**MIC**		**FIC**	**FIC index**
	**AMP (μ** **g/ml)**	**SSE (mg/ml)**	**NIS (μ** **g/ml)**	**AMP + SSE (μ** **g/ml + mg/ml)**	
*S. aureus* DMST 20651	>512^R^	4.0^ND^	16	0.15 + 2.0	<0.5
*S. aureus* DMST 20652	>512^R^	4.0^ND^	16	0.15 + 2.0	<0.5
*S. aureus* DMST 20653	>512^R^	4.0^ND^	16	0.15 + 2.0	<0.5
*S. aureus ATCC 29213	0.25^S^	4.0^ND^	0.5	NT	NT

^S^ = Susceptible; ^R^ = resistant; ^ND^ = No data in CLSI; NT = not test; AMP = Ampicillin; SSE = *S. suberosa* crude extract; NIS = Nisin; FICI ≤ 0.5 denoting synergistic; FICI > 0.5–4.0 denoting no interaction; FICI > 4.0 denoting antagonism; *S. aureus ATCC 29213 was used as a reference strain.

### Killing curve determinations

**Figure 1:**
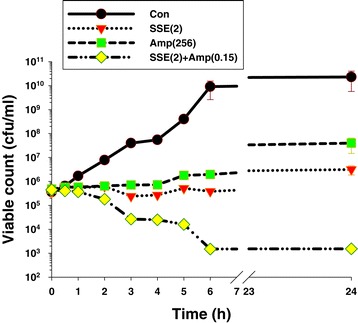
**Time killing-curve of ARSA after exposure to SSE, ampicillin either alone or in combination.** Con = control (drug free); SSE (2) = SSE at 2 mg/ml; Amp(256) = ampicillin at 256 μg/ml; SSE(2) + Amp(0.15) = SSE at 2 mg/ml plus ampicillin at 0.15 μg/ml; the values plotted are the means of 4 observations, and the vertical bars indicate the standard errors of the means.

The viable counts for ARSA after exposure to antimicrobial agents at different times are shown in Figure 1. The control cells revealed no reduction in viable counts and steady growth in log phase viable counts throughout 24 h. Whereas, no significant change was observed in cells treated with the SSE and ampicillin alone. Interestingly, the combination of the SSE plus ampicillin exhibited a steady reduction of 5 × 10^5^ cfu/ml to 10^3^ cfu/ml within 6 h and did not recover within 24 h. These results had also been confirmed antibacterial and synergistic activity of MIC and checkerboard determinations.

### TEM

**Figure 2:**
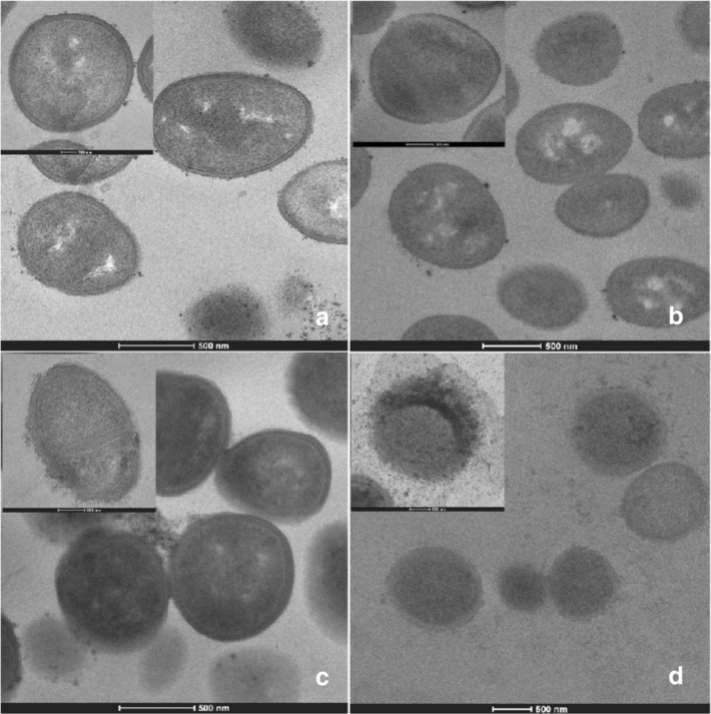
**Ultrathin sections of log phase of ARSA DMST 20651 grown in MHB: a = Control (bar = 500 nm, x19500;** ***inset*** **: bar = 100 nm, x43000); b = 256 μg/ml ampicillin (bar = 500 nm, x15000; inset** **: bar = 200 nm, x38000); c = 2 mg/ml SSE (bar = 500 nm, x19500;** ***inset*** **: bar = 200 nm, x38000); d = 1.5 mg/ml SSE plus 0.11 μg/ml ampicillin (bar = 500 nm, x8700;** ***inset*** **: bar = 200 nm, x29000).**

**Figure 3:**
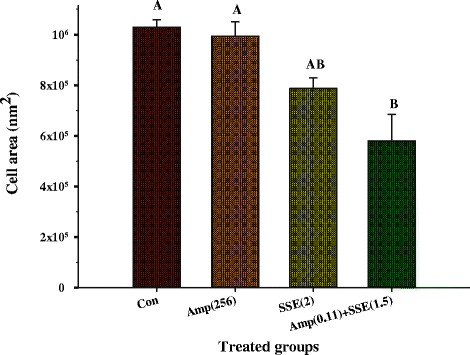
**The cell area of ARSA after treatment with SSE, ampicillin either alone or in combination: Con = control (drug free); Amp (256) = ampicillin at 256 μg/ml; SSE (2) = SSE at 2 mg/ml; Am (0.11) + SSE (1.5) = ampicillin at 0.11 μg/ml plus SSE at 1.5 mg/ml; The graph shows an area of cell determined by cell width x cell length (nm** ^**2**^ **).** The different superscript alphabets are significantly different from each other. Each treated group was compared using one-way ANOVA and Tukey’s HSD Post-hoc test, *p* < 0.01 are presented.

The electron microscope images were chosen to present from triplicate samples in each group. Electron microscopic investigation clearly exhibited that the cytoplasmic membrane and cell wall of ARSA grown in the absence of antibacterial agent (control) can be undoubtedly distinguished and no damage to ultrastructure was observed (Figure 2a). ARSA treated with ampicillin 256 μg/ml alone showed slight peptidoglycan damage to a minority of these cells (Figure 2b). A number of these cells treated with SSE 2 mg/ml caused somewhat peptidoglycan damage (Figure 2c). Besides, these average cell areas were somewhat smaller than the control and ampicillin groups, but not a significant difference (*p* > 0.01) (Figure 3). These findings indicate that the SSE treated cells cause rather higher peptidoglycan damage than ampicillin treated cells. Obviously, the synergistic effect was observed with the combination of ampicillin plus SSE that these cells demonstrated a lot of these cells exhibited marked morphological damage, noticeable peptidoglycan damage (Figure 2d). Obviously, these average cell areas were significantly smaller than control and others (*p* < 0.01) (Figure 3).

### Immunofluorescence staining and confocal microscopy

**Figure 4:**
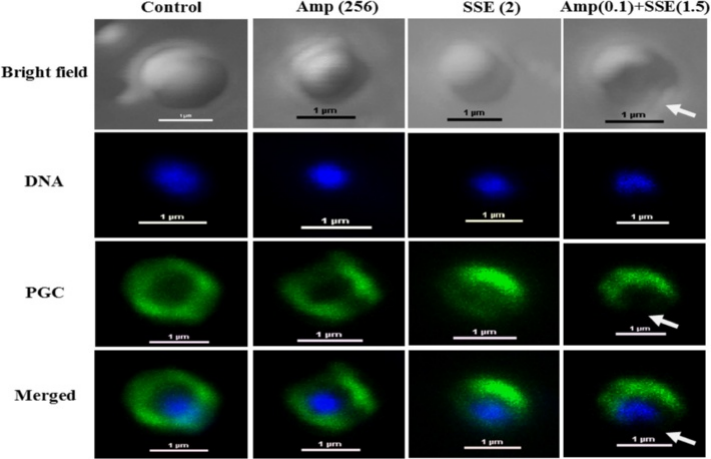
**Schematic representation of the results from immunofluorescence and a confocal laser scanning microscope; Samples of ARSA after treatment for 4 h with Ampicillin, SSE, either alone or in combination.** Amp (256), ampicillin at 256 μg/ml; SSE (2), *Stephania suberosa* extract at 2 mg/ml; Amp (0.1) + SSE (1.5) = Ampicillin 0.11 μg/ml plus SSE 1.5 mg/ml. The cells were stained for DNA with DAPI (blue) and labeled for peptidoglycan (PGC) (green) using respective antibodies. DNA in all groups was localized in the central of cell and surrounded by a peptidoglycan layer (merged images). The white arrows showed explicit disruption of peptidoglycan. Scale bar = 1 μm.

Confocal laser scanning images of peptidoglycan-labeled ARSA unambiguously revealed intact coccus-shaped and no damage was observed in control cell (Figure 4). Cells treated with ampicillin and SSE alone showed a slight damage to peptidoglycan, but SSE alone seemed to have more damage than ampicillin alone. Substantial peptidoglycan disruption was seen in cell received ampicillin plus SSE combination. The bright field image of this treated bacterium demonstrated distortion in cell shape (a white arrow). Data from this experiment had ratified damage of ARSA’s peptidoglycan after treatment with SSE in adjacent with ampicillin. These results support a predominant mechanism of action of this combination probably be inhibiting peptidoglycan synthesis.

### CM permeability

The effect of 256 μg/ml ampicillin, 2 mg/ml SSE alone and the combination of 0.11 μg/ml ampicillin plus 1.5 mg/ml SSE on CM permeability determined by cytoplasmic β-galactosidase activity is illustrated in Table 2. The result showed that there was no activity of β- galactosidase with increasing time in cells grown without antibacterial agent (control), with ampicillin and SSE alone. Whereas, cell treated with SSE plus ampicillin combination and nisin exhibited β-galactosidase activity (observed yellow) after 1 h exposure time. These results indicated that the combination of SSE plus ampicillin revealed the ability to increase CM permeability of ARSA.

**Table 2:** **β**-**galactosidase activity results of ARSA after treatment with ampicillin, SSE alone or in combination**

**Time**	**Control (no drug)**	**Amp (256)**	**SSE (2)**	**Amp + SSE (0.11 + 1.5)**	**NIS (8) (positive control)**
0	Neg	Neg	Neg	Neg	Neg
1	Neg	Neg	Neg	Pos	Pos
2	Neg	Neg	Neg	Pos	Pos
3	Neg	Neg	Neg	Pos	Pos
4	Neg	Neg	Neg	Pos	Pos
5	Neg	Neg	Neg	Pos	Pos

Neg, no evidence of activity; Pos, have evidence of activity; Amp (256), ampicillin at 256 μg/ml; SSE (2), *Stephania suberosa* extract at 2 mg/ml; Amp + SSE (0.11 + 1.5) = Ampicillin 0.11 μg/ml plus SSE 1.5 mg/ml; NIS (8), Nisin at 8 μg/ml was used as a positive control. The experiment was carried out in triplicate observations.

**Figure 5:**
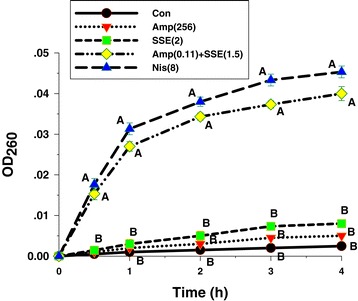
**Presence of 260 nm absorbing material in the supernatants ARSA treated with Ampicillin, SSE, either alone or in combination.** Amp (256), ampicillin at 256 μg/ml; SSE (2), *Stephania suberosa* extract at 2 mg/ml; Amp (0.11 + 1.5) + SSE (1.5) = Ampicillin 0.11 μg/ml plus SSE 1.5 mg/ml; NIS (8), Nisin at 8 μg/ml was used as a positive control and untreated cells were used as negative control. The mean ± SEM for three replicates are illustrated. Means sharing the same superscript are not significantly different from each other (Tukey’s HSD, *p* < 0.01).

Furthermore, the cytoplasmic membrane permeability was measured by UV-absorbing release materials as presented in Figure 5. After treatment ARSA cells with 8 μg/ml nisin, and 0.11 μg/ml ampicillin plus 1.5 mg/ml SSE could induce the release of 260 nm absorbing material, which we interpret to be mostly DNA, RNA, metabolites and ions significantly higher than controls, ampicillin, and SSE alone within 0.5 h and throughout the 4 h (*p* < 0.01). These results imply that the synergistic activity of SSE plus ampicillin increased cytoplasmic membrane permeability of this strain [36,37].

### Enzyme assay

**Figure 6:**
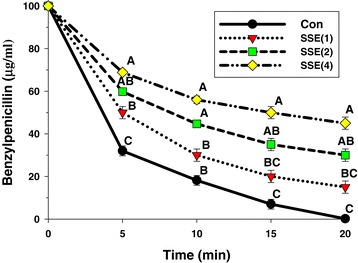
**The inhibitory activity of SSE against** **β-lactamase type IV from** ***E. cloacae*** **in hydrolyzing benzylpenicillin; Con = control (no testing agent); SSE(1) = SSE at 1 mg/ml.** The graph shows the remaining benzylpenicillin at the same time. Means sharing the same superscript are not significantly different from each other (Tukey’s HSD, *p* < 0.01).

The ability of SSE to inhibit activity of β-lactamase type IV isolated from *E. cloacae* was assayed by determining the amount of remaining benzylpenicillin using reverse-phase HPLC. As shown in Figure 6, the result displayed that benzylpenicillin treated with SSE was significantly higher than control starting from 5 minutes (*p* < 0.01). The benzylpenicillin remainder was significantly increased by an increase in SSE as a concentration-dependent manner. These results suggest that one activity of SSE against ARSA may involve in β-lactamase inhibition [9].

## Discussion

The present investigation is the first report of antibacterial and synergistic activities of *S. suberosa* extract when used singly and in combination with ampicillin against clinical isolated ARSA. The preliminary mechanisms of action of those agents were also evaluated in this study. Practically-prescribed antibiotic resistance in MRSA due to drug target-site alteration, enzyme modification and changes in membrane permeability, has increasingly emerged. Therefore, the selection of antibiotic to treat multidrug resistant MRSA has been daily decreasing. So, the research approach to find out new anti-MRSA agents are still necessary [4]. The MIC results revealed that these testing *S. aureus* strains were highly resistant to ampicillin alone because of the standard value of the sensitivity of ampicillin against these strains are ≤ 0.25 μg/ml [27]. As well as, SSE demonstrated little bacteriostatic effect against these strains while the reference *S. aureus* strain exhibits susceptible to ampicillin. Likewise, these results are in substantial agreement with those of Eumkeb et al. [9] that the MIC of ampicillin against *S. aureus* DMST 20651 was > 1,000 μg/ml. Also, the MIC result of nisin against MRSA strains seem consistent with previous finding that 90% MIC of nisin against MRSA was 16 μg/ml [38]. The checkerboard determination revealed synergistic effects of ampicillin plus SSE against all of tested *S. aureus* strains with FIC index at <0.50 [39]. These results are in substantial correspondence with those of Eumkeb et al. that galangin, quercetin or baicalein plus ampicillin exhibited synergistic activity against penicillins-resistant *S. aureus* strains at FIC indices < 0.03 [9]. Besides, previous studies reported that a synergistic effect between quercetin and oxacillin against vancomycin-intermediate *S. aureus* displayed the lowest FIC index value of 0.0417 [40]. Apart from this, the antibacterial activity of quercetin plus ampicillin or vancomycin against the sensitive MRSA strain were significantly increased compared to control (no any testing agent) (*p* < 0.01) [41]. As previously documented, drug combination approach by achieving a synergistic effect can eliminate and neutralize the adverse effects [16]. The killing curve determination can confirm MIC and checkerboard determinations that synergistic effect of SSE plus ampicillin caused marked reduction in viable counts of ARSA cells from 6 h and throughout 24 h. These results appear consistent with previous findings that galangin, quercetin or baicalein plus ceftazidime exhibited synergistic activity against MRSA result in a large decrease from 6 to 24 h [9], Apart from this, the combinations of baicalin and β-lactam antibiotics showed that the killing of MRSA and beta-lactam-resistant *S. aureus* cells were dramatic reduction by these combinations [26]*.* Clearly, the synergistic effect of SSE plus ampicillin against ARSA was observed. For this reason, the elementary mechanism of action such as TEM, CM permeability, and enzyme assay were more investigated.

TEM results of SSE plus ampicillin treated cells demonstrated that ARSA cells exhibited marked morphological damage, clear peptidoglycan and cytoplasmic membrane damage, and average cell areas significant smaller than control. These results seem consistent with previous findings that the combination of ceftazidime plus galangin caused damage to the ultrastructures of the cells, affected the integrity of the cell walls and led to an increase in cell size of ceftazidime-resistant *S. aureus* [9]. The TEM results have been confirmed by confocal microscopic images that the peptidoglycan of this combination treated cells was undoubtedly destroyed. These effects can be explained by assuming that SSE may insert synergistic action with ampicillin to inhibit peptidoglycan synthesis leads to marked morphological damage and delay cell division.

The CM permeability revealed that SSE in combination with ampicillin increased cytoplasmic membrane permeability of this strain. The β-galactosidase activity result was virtually the same as UV-absorbing material concentrations result that CM permeability was significantly increased from 1 h onward (Table 2 and Figure 5). These results are in substantial agreement with previous findings that luteolin either alone or combined with amoxicillin and apigenin alone and in combination with ceftazidime increased CM permeability of amoxicillin-resistant *E. coli* and ceftazidime-resistant *E. cloacae* respectively [15,28]. In general, nisin incorporates into the membrane and makes the membrane permeable for ions. So that, both the membrane potential and pH gradient are dissipated [42]. Apart from this, nisin inhibits peptidoglycan synthesis and forms highly specific pores through interaction with the membrane-bound cell wall precursor lipid II [43]. The increase in CM permeability may be one of the synergistic activity of this combination against ARSA strain. The more SSE concentrations results in more benzylpenicillin remainder of enzyme assay results are in substantial agreement with previous findings that galangin inhibits β-lactamase in a concentration-dependent manner [9]. Noteworthy that the SSE alone could show β-lactamase inhibitory activity results in very high MIC value. Whereas, its combination with ampicillin showed a synergistic effect by peptidoglycan synthesis inhibition and increase CM permeability. Six new protoberberines and ten known alkaloids were found in *Stephama suberosa* root extracts [44]. However, the bioactive compounds of *S. suberosa* extract that showed antibacterial effect in this study have not been well characterized. Although, there is devoid of the report has been documented for toxicity of *S. suberosa*, but some plant in the genus *Stephania*, for instance, previous studies found that the LD_50_ of oral feeding of aqueous extract of *Stephania cepharantha* wet and dry root tuber in mice were 41.4 g/kg and 22.9 g/kg respectively [45]. In addition, the acute toxicities of protoberberine alkaloids, berberine, coptisine, palmatine and epiberberine, from *Rhizoma coptidis* (RC) were evaluated, the LD_50_ value of four alkaloids were 713.57, 852.12, 1533.68 and 1360 mg/kg, respectively. The sub-chronic toxicity study in rats treated with the RC alkaloids at a dose of 156 mg/kg/day for 90 days revealed that no abnormality in clinical signs, body weights, organ weights, urinalysis, hematological parameters, gross necropsy, histopathology, no mortality and morbidity were observed in any of the animals [46]. Likewise, the acute oral LD_50_ of lupanine from *Lupinus angustifolius* in rats was 1464 mg/kg [47]. Our finding found that the total alkaloid content in SSE was approximately 526.27 mg/1 g of dried extract. The FIC for SSE in combination with ampicillin against ARSA was 2 mg/ml, possibly therefore the total alkaloids of 1.05 mg may roughly presented in 2 mg/ml. In this case, the in vitro determination of starting dose for in vivo tests was used to predict starting doses for subsequent in vivo acute lethality assays. The results lend support to the assumption that if most of these alkaloids are Cepharanthine, the estimated LD_50_ was > 5,000 mg/kg, which is classified as practically nontoxic [48,49]. Hence, SSE when used in combination with ampicillin at this concentration may have a sufficient margin of safety for therapeutic use. Obviously, many alkaloids have been used as modern medicine, for example colchicine (anti-gout), quinine (anti-malaria), morphine and codeine (analgesics), reserpine (anti-hypertension), vinblastine and vincristine (anti-cancer), theophylline (anti-asthma) [50,51]. However, further investigation should be focused on active ingredients of SSE that play an important role on antibacterial effect, as well as toxicity confirmation is still necessary.

## Conclusions

In summary, our study provides evidence that SSE has the extraordinary potential to reverse bacterial resistance to originate traditional drug susceptibility of it. This is the first report of the mechanism of synergistic action of SSE plus ampicillin combination against ampicillin-resistant *S. aureus*. Three modes of actions would be implied that this combination inhibit peptidoglycan synthesis, inhibit β-lactamases activity, and increase CM permeability. So, this *Stephania suberosa* proposes the high potential to develop a useful of novel adjunct phytopharmaceutical to ampicillin for the treatment of ARSA. Future studies should be investigated and confirmed the efficacy and toxicity of this combination in an animal test or in humans, Also, The synergistic effect on blood and tissue would be evaluated and achieved.

## Abbreviations

ARSA: Ampicillin-resistant *Staphylococcus aureus* DMST 20651
SSE: *Stephania suberosa* Forman extract
MIC: Minimal inhibitory concentration
FIC: Fraction inhibitory concentration
ATCC: American Type Culture Collection
CM: Cytoplasmic membrane
cfu: Colony forming unit
CAMHB: Cation-adjusted Mueller-Hinton broth
OD: Optical density
MHA: Mueller-Hinton agar
HPLC: High performance liquid chromatography
PBS: Phosphate buffer solution
BSA: Bovine serum albumin
DAPI: 4′,6-Diamidino-2-Phenylindole
CLSI: Clinical and Laboratory Standards Institute
DMSO: Dimethyl sulfoxide

## References

[1] Huttner A, Harbarth S, Carlet J, Cosgrove S, Goossens H, Holmes A, Jarlier V, Voss A, Pittet D (2013). Antimicrobial resistance: a global view from the 2013 World Healthcare-Associated Infections Forum. Antimicrob Resist Infect Control.

[2] Chambers HF (2001). The changing epidemiology of *Staphylococcus aureus*?. Emerg Infect Dis.

[3] Karlsson-Kanth A, Tegmark-Wisell K, Arvidson S, Oscarsson J (2006). Natural human isolates of *Staphylococcus aureus* selected for high production of proteases and alpha-hemolysin are sigmaB deficient. Int J Med Microbiol.

[4] Mun SH, Joung DK, Kim YS, Kang OH, Kim SB, Seo YS, Kim YC, Lee DS, Shin DW, Kweon KT, Kwon DY (2013). Synergistic antibacterial effect of curcumin against methicillin-resistant *Staphylococcus aureus*. Phytomedicine.

[5] Engemann JJ, Carmeli Y, Cosgrove SE, Fowler VG, Bronstein MZ, Trivette SL, Briggs JP, Sexton DJ, Kaye KS (2003). Adverse clinical and economic outcomes attributable to methicillin resistance among patients with *Staphylococcus aureus* surgical site infection. Clin Infect Dis.

[6] Laupland KB, Lyytikainen O, Sogaard M, Kennedy KJ, Knudsen JD, Ostergaard C, Galbraith JC, Valiquette L, Jacobsson G, Collignon P, Schonheyder HC (2013). The changing epidemiology of *Staphylococcus aureus* bloodstream infection: a multinational population-based surveillance study. Clin Microbiol Infect.

[7] Mehta S, Singh C, Plata KB, Chanda PK, Paul A, Riosa S, Rosato RR, Rosato AE (2012). beta-Lactams increase the antibacterial activity of daptomycin against clinical methicillin-resistant *Staphylococcus aureus* strains and prevent selection of daptomycin-resistant derivatives. Antimicrob Agents Chemother.

[8] Jimenez JN, Ocampo AM, Vanegas JM, Rodriguez EA, Mediavilla JR, Chen L, Muskus CE, Velez LA, Rojas C, Restrepo AV, Garces C, Kreiswirth BN, Correa MM (2013). A comparison of methicillin-resistant and methicillin-susceptible *Staphylococcus aureus* reveals no clinical and epidemiological but molecular differences. Int J Med Microbiol.

[9] Eumkeb G, Sakdarat S, Siriwong S (2010). Reversing beta-lactam antibiotic resistance of *Staphylococcus aureus* with galangin from *Alpinia officinarum* Hance and synergism with ceftazidime. Phytomedicine.

[10] Arede P, Milheirico C, de Lencastre H, Oliveira DC (2012). The anti-repressor MecR2 promotes the proteolysis of the *mecA* repressor and enables optimal expression of beta-lactam resistance in MRSA. PLoS Pathog.

[11] Arede P, Oliveira DC (2013). Proteolysis of mecA repressor is essential for expression of methicillin resistance by *Staphylococcus aureus*. Antimicrob Agents Chemother.

[12] Zhang HZ, Hackbarth CJ, Chansky KM, Chambers HF (2001). A proteolytic transmembrane signaling pathway and resistance to beta-lactams in staphylococci. Science.

[13] Chung PY, Navaratnam P, Chung LY (2011). Synergistic antimicrobial activity between pentacyclic triterpenoids and antibiotics against *Staphylococcus aureus* strains. Ann Clin Microbiol Antimicrob.

[14] Hemaiswarya S, Kruthiventi AK, Doble M (2008). Synergism between natural products and antibiotics against infectious diseases. Phytomedicine.

[15] Eumkeb G, Chukrathok S (2013). Synergistic activity and mechanism of action of ceftazidime and apigenin combination against ceftazidime-resistant *Enterobacter cloacae*. Phytomedicine.

[16] Wagner H (2011). Synergy research: approaching a new generation of phytopharmaceuticals. Fitoterapia.

[17] Worthington RJ, Melander C (2013). Combination approaches to combat multidrug-resistant bacteria. Trends Biotechnol.

[18] Wright GD, Sutherland AD (2007). New strategies for combating multidrug-resistant bacteria. Trends Mol Med.

[19] Semwal DK, Rawat U (2009). Antimicrobial hasubanalactam alkaloid from *Stephania glabra*. Planta Med.

[20] Semwal DK, Badoni R, Semwal R, Kothiyal SK, Singh GJ, Rawat U (2010). The genus *Stephania* (Menispermaceae): chemical and pharmacological perspectives. J Ethnopharmacol.

[21] Al-Daihan S, Al-Faham M, Al-shawi N, Almayman R, Brnawi A, Zargar S, Bhat R (2013). Antibacterial activity and phytochemical screening of some medicinal plants commonly used in Saudi Arabia against selected pathogenic microorganisms. J King Saud Univ Sci.

[22] Savithramma N, Rao ML, Suhrulatha D (2011). Screening of medicinal plants for secondary metabolites. Middle-East J Sci Res.

[23] Xavier J, Johnson N (2013). A study on phytochemical, pharmacological and anti-insecticidal activity of *Leucas aspera* (WILLD) Linn. Int J Bio Pharm Allied Sci.

[24] Yadav R, Agarwala M (2011). Phytochemical analysis of some medicinal plants. J Geophys Res.

[25] Pochapski MT, Fosquiera EC, Esmerino LA, Dos Santos EB, Farago PV, Santos FA, Groppo FC (2011). Phytochemical screening, antioxidant, and antimicrobial activities of the crude leaves’ extract from *Ipomoea batatas* (L.) Lam. Pharmacogn Mag.

[26] Liu IX, Durham DG, Richards RM (2000). Baicalin synergy with beta-lactam antibiotics against methicillin-resistant *Staphylococcus aureus* and other beta-lactam-resistant strains of *S. aureus*. J Pharm Pharmacol.

[27] Matthew AW, Franklin RC, William AC, Micheal ND, George ME, David WH, Janet FH, Mary JF, Jana MS, Donal EL, Danie JS, Fred CT, John DT, Melvin PW, Barbara LZ, Clinical Laboratory Standards Institute (2012). **Methods for Dilution Antimicrobial Susceptibility Tests for Bacteria That Grow Aerobically**. *Clinical and Laboratory Standards Institute document M7-A7 Volume* 29.

[28] Eumkeb G, Siriwong S, Thumanu K (2012). Synergistic activity of luteolin and amoxicillin combination against amoxicillin-resistant *Escherichia coli* and mode of action. J Photochem Photobiol B.

[29] Bonapace CR, Bosso JA, Friedrich LV, White RL (2002). Comparison of methods of interpretation of checkerboard synergy testing. Diagn Microbiol Infect Dis.

[30] Odds FC (2003). Synergy, antagonism, and what the chequerboard puts between them. J Antimicrob Chemother.

[31] Richards RM, Xing DK (1993). In vitro evaluation of the antimicrobial activities of selected lozenges. J Pharm Sci.

[32] Richards RM, Xing JZ, Gregory DW, Marshall D (1995). Mechanism of sulphadiazine enhancement of trimethoprim activity against sulphadiazine-resistant *Enterococcus faecalis*. J Antimicrob Chemother.

[33] Tocheva EI, Matson EG, Morris DM, Moussavi F, Leadbetter JR, Jensen GJ (2011). Peptidoglycan remodeling and conversion of an inner membrane into an outer membrane during sporulation. Cell.

[34] Eumkeb G, Siriwong S, Phitaktim S, Rojtinnakorn N, Sakdarat S (2012). Synergistic activity and mode of action of flavonoids isolated from smaller galangal and amoxicillin combinations against amoxicillin-resistant *Escherichia coli*. J Appl Microbiol.

[35] Marri L, Dallai R, Marchini D (1996). The novel antibacterial peptide ceratotoxin A alters permeability of the inner and outer membrane of *Escherichia coli* K-12. Curr Microbiol.

[36] Shen L, Liu D, Li M, Jin F, Din M, Parnell LD, Lai CQ (2012). Mechanism of action of recombinant acc-royalisin from royal jelly of Asian honeybee against gram-positive bacteria. PLoS One.

[37] Zhou K, Zhou W, Li P, Liu G, Zhang J, Dai Y (2008). Mode of action of pentocin 31–1: An antilisteria bacteriocin produced by *Lactobacillus pentosus* from Chinese traditional ham. Food Control.

[38] Giacometti A, Cirioni O, Barchiesi F, Scalise G (2000). In-vitro activity and killing effect of polycationic peptides on methicillin-resistant *Staphylococcus aureus* and interactions with clinically used antibiotics. Diagn Microbiol Infect Dis.

[39] Marques MB, Brookings ES, Moser SA, Sonke PB, Waites KB (1997). Comparative in vitro antimicrobial susceptibilities of nosocomial isolates of *Acinetobacter baumannii* and synergistic activities of nine antimicrobial combinations. Antimicrob Agents Chemother.

[40] Basri DF, Zin NM, Bakar NS, Rahmat F, Mohtar M (2008). Synergistic Effects of Phytochemicals and Oxacillin on Laboratory Passage-Derived Vancomycin-Intermediate *Staphylococcus aureus* Strain. J Med Sci.

[41] Hirai I, Okuno M, Katsuma R, Arita N, Tachibana M, Yamamoto Y (2010). Characterisation of anti-*Staphylococcus aureus* activity of quercetin. Int J Food Sci Tech.

[42] Gao F, Abee T, Konings W (1991). Mechanism of action of the peptide antibiotic nisin in liposomes and cytochrome c oxidase-containing proteoliposomes. Appl Environ Microbiol.

[43] Wiedemann I, Breukink E, van Kraaij C, Kuipers OP, Bierbaum G, de Kruijff B, Sahl H-G (2001). Specific binding of nisin to the peptidoglycan precursor lipid II combines pore formation and inhibition of cell wall biosynthesis for potent antibiotic activity. J Biol Chem.

[44] Patra A, Montgomery CT, Freyer AJ, Guinaudeau H, Shamma M, Tantisewie B, Pharadai K (1987). The protoberberine alkaloids of *Stephania suberosa*. Phytochemistry.

[45] Chen J, Tong Y, Zhang X, Tian H, Chang Z (1999). Acute toxicity of *Stephania cepharantha*. hong Yao Cai.

[46] Yi J, Ye X, Wang D, He K, Yang Y, Liu X, Li X (2013). Safety evaluation of main alkaloids from *Rhizoma Coptidis*. J Ethnopharmacol.

[47] Petterson D, Ellis Z, Harris D, Spadek Z (1987). Acute toxicity of the major alkaloids of cultivated *Lupinus angustifolius* seed to rats. J Appl Toxicol.

[48] National institute of Environmental Health Sciences (2001). *Guidance document on using in vitro data to estimate in vivo starting doses for acute toxicity*.

[49] Kennedy GL, Ferenz RL, Burgess BA (1986). Estimation of acute oral toxicity in rates by determination of the approximate lethal dose rather than the LD50. J Appl Toxicol.

[50] Bruneton J (1999). Pharmacognosy, Phytochemistry, Medicinal Plants.

[51] Brunton L, Chabner B (2011). Goodman and Gilman’s The Pharmacological Basis of Therapeutics.

